# The importance of decision bias for predicting eyewitness lineup choices: toward a Lineup Skills Test

**DOI:** 10.1186/s41235-018-0150-3

**Published:** 2019-01-28

**Authors:** Mario J. Baldassari, Justin Kantner, D. Stephen Lindsay

**Affiliations:** 1 Department of Psychology, University of Victoria, PO Box 1700, STN CSC, Victoria, BC V8W2Y2 Canada; 2 Department of Psychology, California State University, Northridge, 18111 Nordhoff Street, Northridge, CA 91330 USA

**Keywords:** Memory, Eyewitness, Recognition, Response bias, Individual differences, Face recognition, Lineup

## Abstract

**ᅟ:**

We report on research on individual-difference measures that could be used to assess the validity of eyewitness identification decisions.

**Background:**

The predictive utility of face recognition tasks for eyewitness identification has received some attention from psychologists, but the previous research focused primarily on witnesses’ likelihood of correctly choosing the culprit when present in a lineup. Far less discussed has been individual differences in witnesses’ proclivity to choose from a lineup that does not contain the culprit. We designed a two-alternative non-forced-choice face recognition task (consisting of mini-lineup test pairs, half old/new and half new/new) to predict witnesses’ proclivity to choose for a set of culprit-absent lineups associated with earlier-viewed crime videos.

**Results:**

In two studies involving a total of 402 participants, proclivity to choose on new/new pairs predicted mistaken identifications on culprit-absent lineups, with *r* values averaging .43. The likelihood of choosing correctly on old/new pairs (a measure of face recognition skill) was only weakly predictive of correct identifications in culprit-present lineups (mean *r* of .22).

**Conclusions:**

Our findings could be the basis for further research aimed at developing a standardized measure of proclivity to choose that could be used, along with other measures, to weigh eyewitnesses’ lineup identification decisions.

**Electronic supplementary material:**

The online version of this article (10.1186/s41235-018-0150-3) contains supplementary material, which is available to authorized users.

## Significance statement

Hundreds of former prison inmates have been exonerated following mistaken criminal convictions partly based on incorrect eyewitness identification evidence. Many published papers have shown that jurors and judges are surprisingly poor at assessing the quality of a witness’s memory, usually placing too much faith in testimony that is error-laden and subject to predictable biases. We present studies conducted as part of an effort to develop a test of the likelihood that a particular witness will make a correct decision when judging a police lineup. We developed the Lineup Skills Test (LST) of both (a) a person’s ability to discriminate previously seen from new faces presented in pairs and (b) a person’s proclivity to mistakenly choose from pairs of new faces. Our results indicate that witnesses’ scores on this skills test are correlated with their responses on full-sized lineups for the culprits of earlier-viewed crime videos. Our test was better at predicting mistaken identifications on culprit-absent (CA) lineups than it was at predicting accurate identifications on culprit-present (CP) lineups.

## Background

Individual differences may predispose some people to be more likely than others to make accurate eyewitness identification decisions. Indeed, such differences have been in the hive mind of psychologists since Munsterberg (1908/2009, p. 47) first published *On the Witness Stand* at the beginning of the twentieth century: “The courts will have to learn, sooner or later, that the individual differences of [people] can be tested to-day by the methods of experimental psychology far beyond anything which common sense and social experience suggest”. Our aim in the current project is to contribute to the development of useful measures of an eyewitness’s lineup identification skill, both in terms of their ability to identify the culprit when present in a lineup (sensitivity) and their ability to reject a lineup when the culprit is not present (proclivity to choose).

Psychologists have reported many studies of suspect identification in which witnessing conditions, delay, and/or testing conditions were systematically manipulated (see Granhag, Ask, & Giolla, 2014; Valentine, 2014, for reviews). However, many studies have shown varying levels of performance in identification tasks among participants, even when all had comparable encoding, delay, and testing conditions (Darling, Martin, Hellmann, & Memon, 2009; Valentine, Pickering, & Darling, 2003). Beyond other known causes for differences in unfamiliar face recognition accuracy (e.g., age, gender, and race) and random measurement errors, these variations in performance likely reflect individual differences in both (a) their skill at encoding, retaining, and identifying target faces amongst distractors and (b) their response bias or proclivity to choose (Kantner & Lindsay, 2012; Megreya & Burton, 2007). If face recognition sensitivity and response bias are stable individual differences, measures of face recognition ability and face memory response bias should be reliable predictors of eyewitness identification (ID) skill.^1^

The literature on the Cambridge Face Memory Test (CFMT), which was developed to diagnose prosopagnosia by Duchaine and Nakayama (2006), provides a basis for expectations regarding the size of the correlations between face recognition tasks and lineup tasks. In the CFMT, the participant first memorizes a face seen from three angles for 3 s each and then attempts to choose that face from 3 three-alternative forced-choice trials varying in viewing angle and using the same image as was studied. After this procedure is repeated for five other faces, participants study all six faces at the same time in a frontal view for 20 s and then are tested for any of the six from a set of 30 three-alternative forced-choice trials containing new images of faces studied in the first phase. The test finishes with another study phase of all six faces at once and 24 more trials of novel photos with the faces slightly obscured by Gaussian noise. The reliability of the CFMT is well established, both originally by Duchaine and Nakayama (2006) and in many studies since. Internal reliability scores within and correlations between two variations of the CFMT (traditional CFMT and new CFMT-Aus, McKone et al., 2011) indicated a theoretical upper bound of *r* = .86, based on a measured *r*(72) = .61 (see Table 1 for details).

**Table 1:** Literature measuring correlation with the Cambridge Face Memory Test

Paper	Predictor	*r*	*N*	CI lower	CI upper
Bobak et al., 2016	Face-matching HR	0.61^a^	27	0.29	0.8
	Face-matching FAR	0.57^a^	27	0.24	0.78
	Face memory target-present trials	0.38^a^	27	0	0.67
	Face memory target-absent trials	0.46^a^	27	0.1	0.72
Bowles et al., 2009	CFPT	0.61	124	0.24	0.8
McGugin et al., 2012	Holistic processing test	0.26	109	0.09	0.44
McKone et al., 2011	CFMT-Aus	0.61	74	0.44	0.74

Where not reported, 95% CIs calculated using vassarstats.net/rho.html

*CI* confidence interval, *CFMT-Aus* Cambridge Face Memory Test (Australia), *CFPT* Cambridge Face Perception Test, *FAR* False alarm rate, *HR* Hit rate

^a^Spearman’s rho calculated by authors, used here as well

Scores on the CFMT have been thoroughly examined for correlation with related measures, as shown in Table 1 (Bobak, Hancock, & Bate, 2016; Bowles et al., 2009; McGugin, Richler, Herzmann, Speegle, & Gauthier, 2012). The large fluctuations in the strength of the relationships between these seemingly very similar tasks leaves the possible upper bound of these correlations (and indeed perhaps the test/retest reliability of the lineup measures) an open question. However, a measure of face memory that is predictive of lineup performance with the strength of most of the larger relationships found in the CFMT literature (*r* = .6) could serve as the basis for a measure useful for real-world policing in assessing the quality of eyewitness IDs. In the current work, we test the importance of knowing an individual’s proclivity to choose for such a measure.

Individual differences in face recognition ability have been used as a predictor of lineup identification accuracy with some success, though few researchers have found relationships stronger than *r* = .4. In the following we briefly summarize all the published studies of which we are aware that explored the relationship between sensitivity or response bias on tests of face recognition and performance in CP or CA lineup identification tasks. The sample sizes in many of the individual studies were small, but they collectively encourage optimism regarding the prospect of developing face recognition tests that usefully inform assessments of individual witnesses’ accuracy on lineups.

Hosch (1994) reported the first data of this kind in which participants’ scores on the Benton Facial Recognition Test (BFRT), which is a face-matching task that was the standard for prosopagnosia testing at the time, were significantly correlated with accuracy on a lineup.^2^ (See Table 2 for *r* values, sample sizes, and 95% confidence intervals [CIs] around *r*.) This correlation varied around *r* = .45 across three small-*N* studies with slightly different procedures, but two other studies using the BFRT did not produce correlations larger than *r* = .05. Using two new samples, Hosch tested the relationship between accuracy on the same lineup task and measures of sensitivity and response bias on a yes/no face recognition task. The number of trials in the face task was not reported, but the first sample yielded no correlation between sensitivity and ID accuracy and a significant correlation between response bias and ID accuracy. Also, participants who produced a correct selection on a CP lineup were more conservative in their face recognition decisions (*B″* mean = .59) than those who produced a false alarm on a CA lineup (*B″* mean = −.1). A second study weakly replicated these findings (see Table 2). The samples in Hosch’s studies were not large enough to produce a stable estimate of the correlation strength (Schönbrodt & Perugini, 2013). Nonetheless, these data provided evidence that face recognition scores can predict eyewitness identification accuracy.

**Table 2:** Literature measuring correlation for lineup accuracy

Paper	Predictor	Outcome	*r*	*N*	CI lower	CI upper
Andersen et al., 2014	CFMT	CP simultaneous lineup	0.26^a^	119	0.09	0.42
	CFMT	CA simultaneous lineup	0.28^a^	119	0.1	0.44
	CFMT	CP sequential lineup	ns^b^	119		
	CFMT	CA sequential lineup	0.27^a^	119	0.09	0.43
Bindemann et al., 2012	Hit rate, Bruce 1-in-10 as memory task	Probability of being a good witness (choosers)	0.7	37	0.49	0.83
	Hit rate, Bruce 1-in-10 as memory task	Probability of being a good witness (choosers)	0.83	86	0.75	0.89
	FA rate, Bruce 1-in-10 as memory task	Probability of being a good witness (nonchoosers)	0.49	43	0.22	0.69
	FA rate, Bruce 1-in-10 as memory task	Probability of being a good witness (nonchoosers)	0.38	99	0.2	0.54
Deffenbacher et al., 1978	Y/N face recognition overall accuracy	4-person simultaneous lineup of class exam administrators	−0.28	45	−0.53	0.01
Hosch, 1994	BFRT	Single lineup of experimenter (half CP)	0.54	32	0.24	0.75
	BFRT	Single lineup of experimenter (half CP)	0.39	38	0.08	0.63
	BFRT	Single lineup of experimenter (half CP)	0.41	27	0.04	0.68
	Y/N face recognition sensitivity	Single lineup of experimenter (half CP)	−0.07	33	−0.4	0.28
	Y/N face recognition sensitivity	Single lineup of experimenter (half CP)	−0.21	36^c^	− 0.5	0.13
	Y/N face recognition response bias	Single lineup of experimenter (half CP)	0.5	33	0.19	0.72
	Y/N face recognition response bias	Single lineup of experimenter (half CP)	0.28	36^c^	−0.05	0.56
Kantner & Lindsay, 2014	Y/N face recognition response bias	1 CP and 4 CA lineups	0.29	65	0.06	0.5

*BFRT* Benton Facial Recognition Task, *CA* culprit absent, *CFMT* Cambridge Face Memory Test, *CI* confidence interval, *CP* culprit present, *FA* False alarm

^a^Chi-squared values converted to correlation coefficients at campbellcollaboration.org/escalc/html/EffectSizeCalculator-R5.php

^b^Non-significant chi-squared value not reported in manuscript

^c^Sample sizes not reported, but are inferred based on reported *p*-values

In a replication of Hosch’s studies, Geiselman et al. (2001) found that participants who chose the culprit from either of two CP lineups tended to have higher scores on the short form of the BFRT. The scores were not predictive on easier lineups in which most participants chose the culprit. Because the difficult lineups used by Geiselman et al. likely mimic those used in the real world (Wells et al., 1998), it seems likely that a face recognition test such as the BFRT could be useful in predicting lineup accuracy when the culprit is present. However, Geiselman et al. did not measure the predictive utility of witness response bias. Additionally, caution has been advised in interpreting the results of experiments using the BFRT, as there is evidence that participants can ignore face identities and still score highly on the BFRT by focusing on eyebrows (Duchaine & Nakayama, 2004).

Morgan et al. (2007) provided evidence of a relationship between face recognition test performance and eyewitness ID in a stressful realistic setting. These researchers observed a positive relationship between face recognition ability and eyewitness accuracy in a group of 46 army trainees. The trainees underwent a stressful interrogation, and later were asked to identify the interrogator from a 10-person sequential lineup. Altogether, 27 participants saw a CP lineup, while the rest saw a CA lineup. Participants’ accuracy on CP lineups was predicted by scores on the face subtest of the Weschler Intelligence Test. This relationship was driven by the tendency for trainees who made a correct decision on the lineup to have produced both fewer false negatives and more true positives in the Weschler test (MANOVA *p*’s < .01). Follow-up analyses demonstrated that participants who produced false positive IDs drove the effect, as this group tended to make fewer true positive responses and more false negatives in the Weschler test (*p*’s between .1 and .05). That false positives drove Morgan et al.’s effects provide evidence that proclivity to choose on a lineup is a predictable individual difference.

Data from Kantner and Lindsay (2012, 2014) indicated that individual differences in rate of calling items studied in a face recognition task may be sufficiently large and reliable to be useful in evaluating eyewitness ID decisions. Several studies have produced evidence of stable trait-like differences in old/new recognition memory response bias across face, word, and painting stimuli and across testing contexts. Kantner and Lindsay (2014) also observed a statistically significant correlation between response bias in a yes/no recognition test with face stimuli and number of IDs made on a set of CA lineups (Table 2).

Bindemann, Brown, Koyas, and Russ (2012) used an altered version of a face-matching task designed by Bruce et al. (1999) to predict lineup performance. Bindemann et al. had participants study target faces and then presented a 10-person test array. Participants who made a correct ID from a CP lineup tended to have higher hit rates on the Bruce test than did participants who had not made a correct ID (reported Cohen’s *d* = .71, our calculated 95% CI [.05, 1.59]; see Table 2 for correlations). Participants who correctly rejected a CA lineup tended to have higher correct rejection rates in the Bruce test than those who chose from a CA lineup (*d* = .93, 95% CI [.26, 1.63]). In a second experiment, participants who made a correct lineup response (either choosing or rejecting) tended to have higher correct rejection rates on the modified Bruce task (choosers’ *d* = .42 [.003, 1.07]; nonchoosers *d* = .54 [.12, .98]). That an individual witness’s proclivity to choose (i.e., response bias) on a lineup was predicted by their proclivity to choose in the modified version of the Bruce task makes sense because the latter is much like a 10-person lineup. However, that a witness’s tendency to choose correctly from a CP lineup was also predicted by their proclivity to choose in the Bruce task (replicating some of Hosch’s 1994 findings) suggests a role for response bias in predicting lineup decisions. That said, the CIs around both effect size estimates were very large and require replication with larger samples.

Andersen, Carlson, Carlson, and Gronlund (2014) measured both face recognition skill (i.e., sensitivity) and proclivity to choose from a lineup by inserting multiple predictors into four separate logistic regressions for simultaneous and sequential CP and CA lineups. Each of their 238 participants watched two videos and saw one CP and one CA lineup. One predictor was participants’ score on the CFMT. Odds ratios indicated that for every unit increase in CFMT score (ranging from 0 to 100), there was a 1% higher likelihood of a correct simultaneous lineup ID, and a 1% lower likelihood of a simultaneous or sequential false positive ID (see Table 2 for correlations derived from a logistic regression). Thus, Anderson et al. supported the hypothesis that the predictive utility of face recognition for identification tasks can be two-sided, in that witnesses showed individual differences in face recognition skill and in proclivity to choose.^3^

Consistent with the idea that performance on face recognition tasks is likely related to performance on suspect ID tasks, some applied researchers use face recognition tasks as proxies for lineups when testing new methods. Weber and colleagues have used mini-lineups with four members as methodological stand-ins for full lineups (e.g., Weber & Varga, 2012). In Weber and Varga’s test of a new lineup procedure, participants studied a list of labelled faces and then were asked to identify a specific studied face (based on the label) out of a lineup of four faces. Responses to these mini-lineups were compared to another set of mini-lineups presented slightly differently (e.g. simultaneous vs. sequential presentation, as in Weber & Brewer, 2004). In other studies, mini-lineups were used to pre-test a theory that was later tested with a traditional video-lineup paradigm with six-person lineups (Sauer, Brewer, & Weber, 2008). This approach implies that a procedure yielding higher accuracy for mini-lineups will also work for full-sized lineups. This assumption seems reasonable, but to the best of our knowledge there is no direct exploration of the relationship between mini-lineups and six-person photospread lineups in the published literature. The current research provided such tests.

As stated by Megreya and Burton (2007), any test measuring the extent to which a witness is good at faces should assess both (a) the witness’s ability to choose correctly from a CP array (face recognition skill) and (b) their ability to correctly reject a CA array (proclivity to choose). The literature reviewed above supports the idea that it may be possible to develop standardized tests of face recognition skill and of proclivity to choose that are sufficiently robust and precise to be of real-world use, but no study has yet produced correlations near the upper bounds suggested by the CFMT data in Table 1 (apart from the low-*N* findings by Hosch, 1994). Moreover, the CFMT and BFRT may not be optimal indices of eyewitness skill. After all, these measures were not initially developed for this use and were intended to diagnose prosopagnosia by assessing sensitivity in face recognition, not response bias. The reviewed literature strongly suggests that proclivity to choose is as predictive of lineup decisions as face recognition skill, yet because investigators have focused mainly on accuracy there are no current tasks developed specifically to predict proclivity to choose. We aimed to fill that need with a test that would be simple to administer, include a substantial number of observations for each construct, closely mimic the presentation of lineups themselves, and involve no deception. To that end, we crafted a preliminary new procedure that we have dubbed the Lineup Skills Test (LST). The long-term ambition of this line of research is to develop a standardized test of eyewitnesses that assesses both (a) a person’s ability to recognize a culprit’s face when it is present in a lineup and (b) a person’s proclivity to choose an innocent suspect when the culprit is absent from a lineup.

## Experiment 1

Participants first studied a large set of faces presented one at a time. The subsequent LST utilized a two-alternative non-forced-choice recognition task (meaning that participants could reject test pairs as unstudied) in which 50% of the trials contained a studied face and an unstudied face and the other 50% contained two unstudied faces.^4^ In essence, each LST trial is a two-person lineup. By measuring accuracy on pairs containing one studied face and one non-studied face (target-present pairs), the LST provides a sensitivity-type measure of face recognition skill. By measuring rejection rates of pairs containing two non-studied faces (target-absent pairs), it provides a measure of proclivity to choose.

In Experiment 1, we tested the extent to which accuracy and proclivity to choose on the LST predict accuracy and proclivity to choose on CA and CP lineups. Participants first viewed a series of crime videos, completed the LST, and then judged a lineup pertaining to each of the videos viewed earlier. To gain more stable estimates of individual decision tendencies on lineups while not including so many lineups that participants would confuse which lineups were associated with which crime videos, we presented five videos during the initial phase of the experiment and the five corresponding lineups at the end. To maximize the number of observations of each type of lineup for a given participant, the presence of the culprit was manipulated between subjects. Thus, half of participants viewed five CA lineups (CA condition), while the other half viewed five CP lineups (CP condition).

### Method

#### Participants

Participants were recruited online via Amazon’s Mechanical Turk (MTurk; CP condition *N* = 122, CA condition *N* = 143) for $0.60. Following exclusionary criteria established before data collection began, participants who confessed to major distractions or to skipping portions of the procedure were removed before we analyzed the data (CP *N* = 8, CA *N* = 12), as were participants who did not stay on the video pages long enough to watch them (CP *N* = 20, CA *N* = 38). Participants who recognized an actor from the video clip were also removed from the CA condition (*N* = 2). Data from the remaining 185 participants were used for analysis.

Participants self-reported their demographics. For the CA condition (*N* = 91), the average reported age was 35 years, with a range from 20 to 66. The sample included 60 women, 69 native English-speakers, 44 who reported having earned at least a bachelor’s degree, and 46 who reported having taken no university courses in psychology. In the CP condition (*N* = 94), the average reported age was 34 years, with a range from 21 to 70. The sample included 54 women, 73 native English-speakers, 67 who reported having earned at least a bachelor’s degree, and 45 who reported having taken no university courses in psychology.

#### Materials

**Fig. 1:**
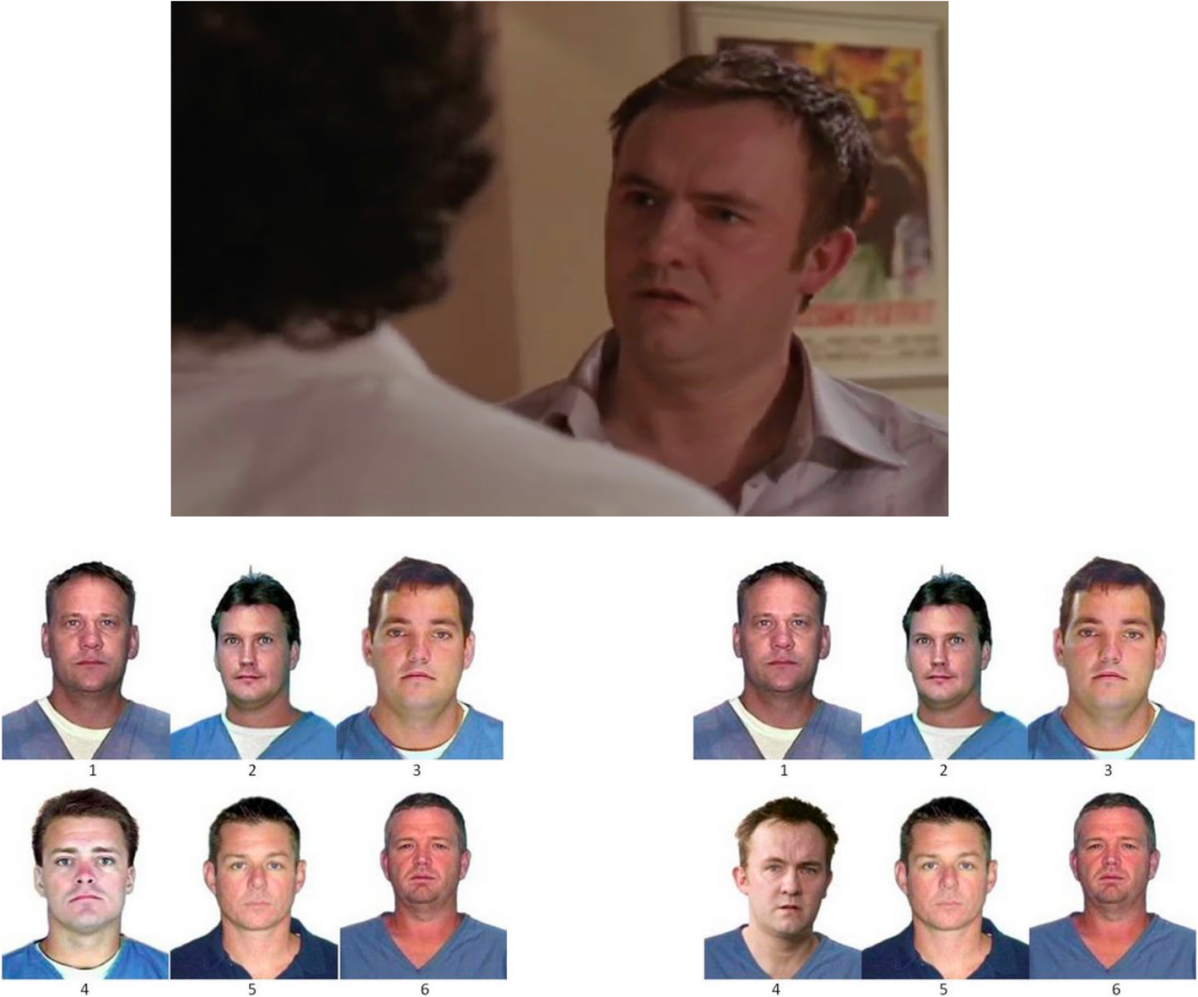
Examples of crime video and lineup materials. The best view of the criminal in the video is included, along with culprit-absent (left) and culprit-present (right) lineups

The five crime videos were clipped from British television crime dramas and depicted middle-aged Caucasian male culprits committing crimes (see the Wiki section of https://osf.io/euchx/ for more information about the videos). Clips ranged from 47 to 83 s in length and were presented with the original sound tracks. The lineups each contained six individual photos about 250 × 350 pixels in size. Figure 1 shows examples of a CA lineup and a CP lineup, which consisted of men who fit a description of the culprit selected from the State of Florida’s online database of criminal mugshots. The photos were edited so that all members were wearing similar clothing. Similar excerpts from the same crime shows were used in unpublished experiments conducted as part of an undergraduate thesis at the University of Victoria by Byrona Tweedy (2011) under the supervision of the third author. We pre-designated as our innocent suspect the member of each CA lineup who had most often been selected in Tweedy’s studies.

For half of the participants, all five lineups were CA, while for the other half, all five contained the culprit. The photo of the culprit in the CP lineup was a still from a portion of the video not included in the video clip presented in the study phase, and the photo was edited so that the criminal was wearing clothes like those of the rest of the lineup members.

The photos for the face test were taken in front of a gray backdrop and showed head-and-shoulders views in color with a neutral expression. Photos were 600 × 600 pixels on screen, and all the people in the photos had no obviously distinctive features such as tattoos or scars. Faces were taken from our in-house face database.^5^ The stimulus set contained 120 Caucasian faces (33 female). For the test phase, we gathered photos taken in the same session as those in the study phase but with the subject smiling (such that face recognition was tested rather than photo recognition; Bruce & Young, 1986).

#### Procedure

MTurk participants accepted the task on Amazon’s work exchange server and were linked to a survey hosted on Qualtrics, where they viewed the crime videos.^6^ Next, participants studied a set of 30 digital photos of Caucasian faces for 1 s each with a 1 s gray mask between. Five pre-randomized photo sets were created such that they all contained differently ordered faces in the LST and a unique rotation of the order in which the five crimes were presented (see Mansour, Beaudry, & Lindsay, 2017 for a discussion of the ecological validity of presenting multiple crimes and lineups). Participants were simply told to watch the videos and were not warned beforehand that the videos would depict crimes. After a 5-min distractor task, participants began our LST test phase. We correctly informed participants that the LST was intended to assess their eyewitness ID skills.

The LST instructions explained the procedure in full and noted that the study had to do with eyewitness ID (see the Appendix). After the study phase, participants moved on to the test, in which a pair of digital photos (450 × 450 pixels each) of faces appeared to the right and left of the mid-point of the screen in each of 60 trials. Half of the trials consisted of one studied or “old” face and one unstudied or “new” face. These constituted the face recognition skill portion of the test, in which the correct answer was either right or left. The other 30 trials each consisted of two unstudied faces. These constituted the proclivity to choose portion of the test, in which the correct answer was neither. The two types of trials were randomly mixed. The first two and last two faces in the study list were not used in the test to avoid primacy and recency effects. Test trials displayed selection options of “Left,” “Neither,” and “Right” that required a mouse click. Participants then rated their confidence for each response on an 11-point scale (0–100). We then reminded participants of our aim to develop a test of lineup skills and emphasized that a good witness chooses the criminal if they are present but also rejects a lineup from which the criminal is absent. Participants finished the procedure by completing five CA or five CP lineups. Crime and lineup order were counterbalanced, and the face recognition study and test phases were presented in a fixed random order that was different for each version of the counterbalance.

### Results

We converted individual accuracy rates on both the LST and the lineups themselves to *z*-scores to facilitate comparison between studies, as they had varying delay lengths, different filler tasks, and different grand average accuracy rates. This practice does not inflate correlation coefficients. In fact, it tends to reduce them slightly. We changed perfect scores and scores of 0 to 1/2 the distance to the next possible score to enable *z*-scoring. See Table 3 for descriptive statistics of raw accuracy scores and [osf.io/euchx/] for participant average data. *z*-scores were created using the NORMSINV function in Microsoft Excel, which returns a standardized value based on the inverse of the raw value (hence the correction for values of 0 and 1). The data are left in their raw form in graphs for readability.

**Table 3:** Descriptive statistics of LST and lineup accuracy

Experiment	LST mean accuracy (SD)			Lineup mean accuracy (SD)			
	New/new	Old/new	*N*	Culprit absent	*N*	Culprit present	*N*
Pilot 1	0.48 (0.26)	0.55 (0.15)	65	0.35 (0.21)	65		
1	0.52 (0.24)	0.60 (0.15)	185	0.43 (0.25)	91	0.54 (0.24)	94
Pilot 2	0.56 (0.21)	0.60 (0.15)	76	0.37 (0.22)	76		
2	0.55 (0.18)	0.56 (0.14)	221	0.41 (0.20)	115	0.40 (0.21)	106

*LST* Lineup Skills Test, *SD* standard deviation

**Fig. 2:**
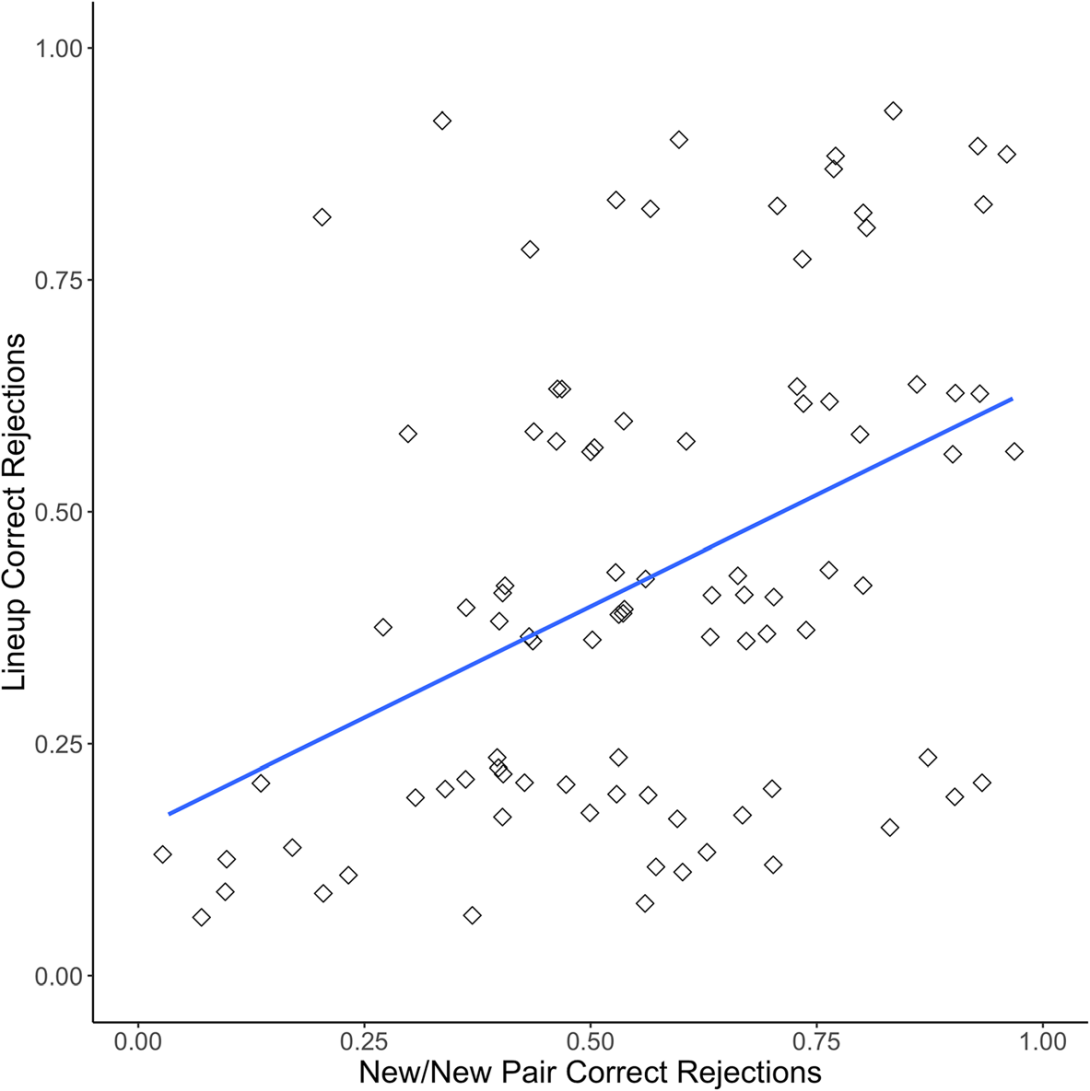
Proclivity to choose correlation for Experiment 1 with linear trendline, both axes jittered

**Fig. 3:**
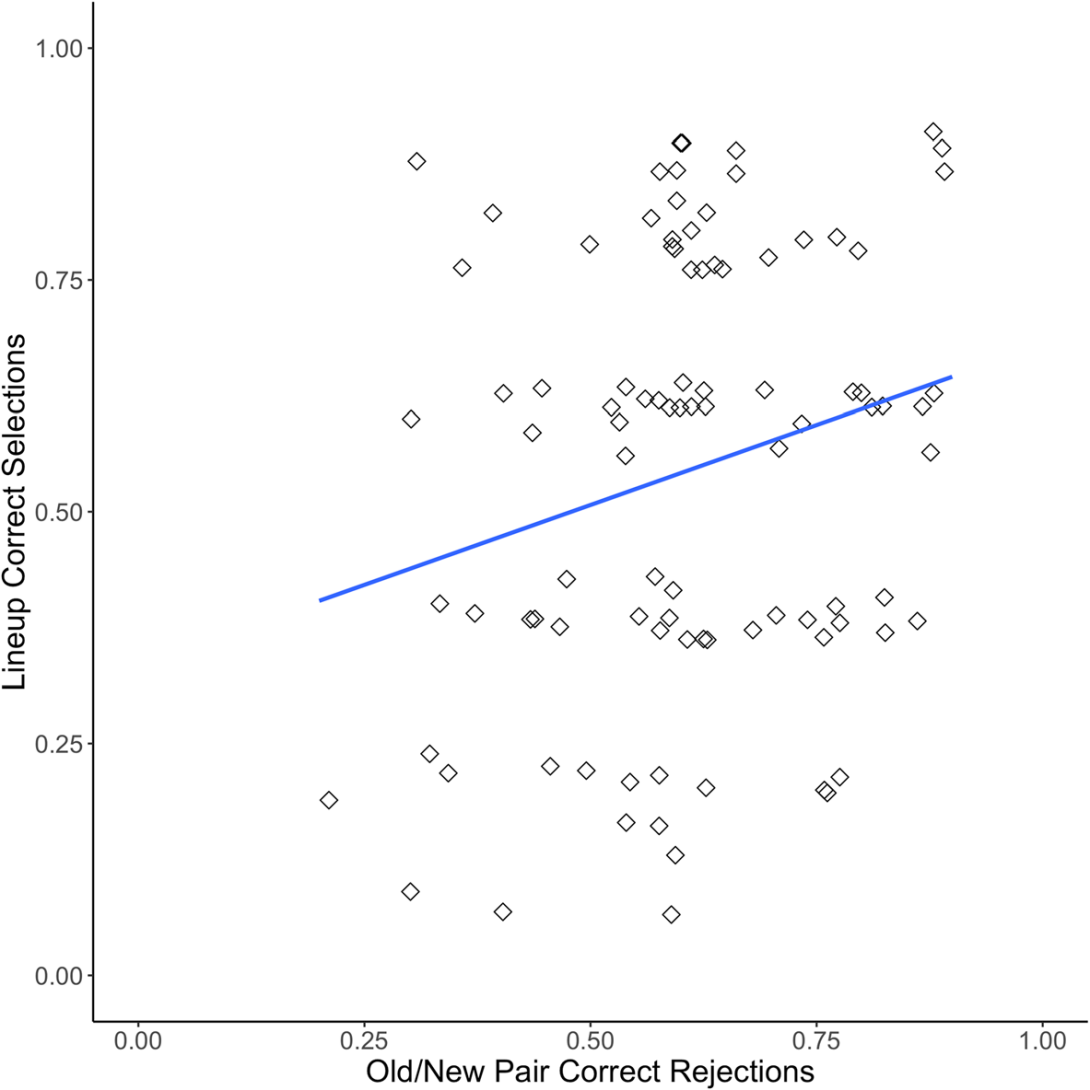
Face recognition skill correlation for Experiment 1 with linear trendline, both axes jittered

Figure 2 displays a jittered scatterplot of the proportion correct for new/new pairs and the proportion correct in CA lineups for Experiment 1. This correlation was significant (*r*(89) = .45, *p* < .001, 95% CI [.27, .60]). There was also a significant correlation between the old/new pair rejection rate and the CA lineup rejection rate (*r*(89) = .58, *p* < .001, 95% CI [.43, .70]). Figure 3 is a jittered scatterplot displaying the overall proportion correct for old/new pairs and the proportion correct in CP lineups (*r*(92) = .22, *p* = .027, 95% CI [.02, .40]).^7^

### Discussion

As predicted, participants who falsely chose more often on new/new pairs in the LST also tended to choose falsely more often on later CA lineups than participants who correctly rejected more new/new pairs. The correlation for this proclivity to choose was of a reasonable size for effects of this type, but it fell short of the larger correlation coefficients of some of the CFMT studies in Table 1. It was, however, larger than the relationship between yes/no face recognition response bias and proclivity to choose on lineups found by Kantner and Lindsay (2014). Unlike expectations based on the findings of Bindemann et al. (2012) and Morgan et al. (2007), old/new pair accuracy was only weakly predictive of CP lineup accuracy.

Having found evidence that the LST can predict performance on lineup ID tasks, we next sought to increase the real-world utility of the test. The police often cannot conduct a lineup on the same day as a crime, as was done in Experiment 1, and the police probably would not want to expose witnesses to many new faces before showing them a lineup. With this in mind, we designed Experiment 2 to include a 2-day delay between exposure to the crime videos and the ID task, and to have participants complete the lineup skills test after, rather than before, the lineups.

## Experiment 2

The purpose of Experiment 2 was to test for relationships between face recognition skill and proclivity to choose with a 2-day delay between the viewing of the crime and the administration of the lineup to make the process more realistic. In addition, we addressed two limitations of the first experiment. First, all the faces were re-randomized into a new set for each participant, thereby controlling for the possibility of effects based purely on our pre-randomized sets. Second, MTurk workers have widely varying internet connection speeds and are sometimes distracted, which may have added error variance to Experiment 1. We conducted Experiment 2 in the lab with undergraduates using E-Prime 2.0.10.242 (2012).

### Methods

#### Participants

Participants (*N* = 221) were recruited via the University of Victoria’s psychology participation pool. They were compensated with extra credit in a psychology course.

#### Materials and procedure

The stimuli were the same as in Experiment 1 except that assignment of faces to condition was randomized anew for each participant. Participants were tested in groups of from 2 to 25. They viewed the five crime videos on a data projector screen. The order of the videos was varied across groups such that each crime was in each position for approximately 1/5 of participants. After the fifth crime video was shown, the participants were dismissed with instructions to return in 2 days. At the beginning of the second session, the lineups were presented with the same title and in the same order as the videos had been presented. Approximately half of the participants viewed all CA lineups (*N* = 115) and the other half viewed all CP lineups (*N* = 106). Immediately after the last lineup, the LST was introduced. The faces in the LST were re-randomized anew for each participant.

### Results

**Fig. 4:**
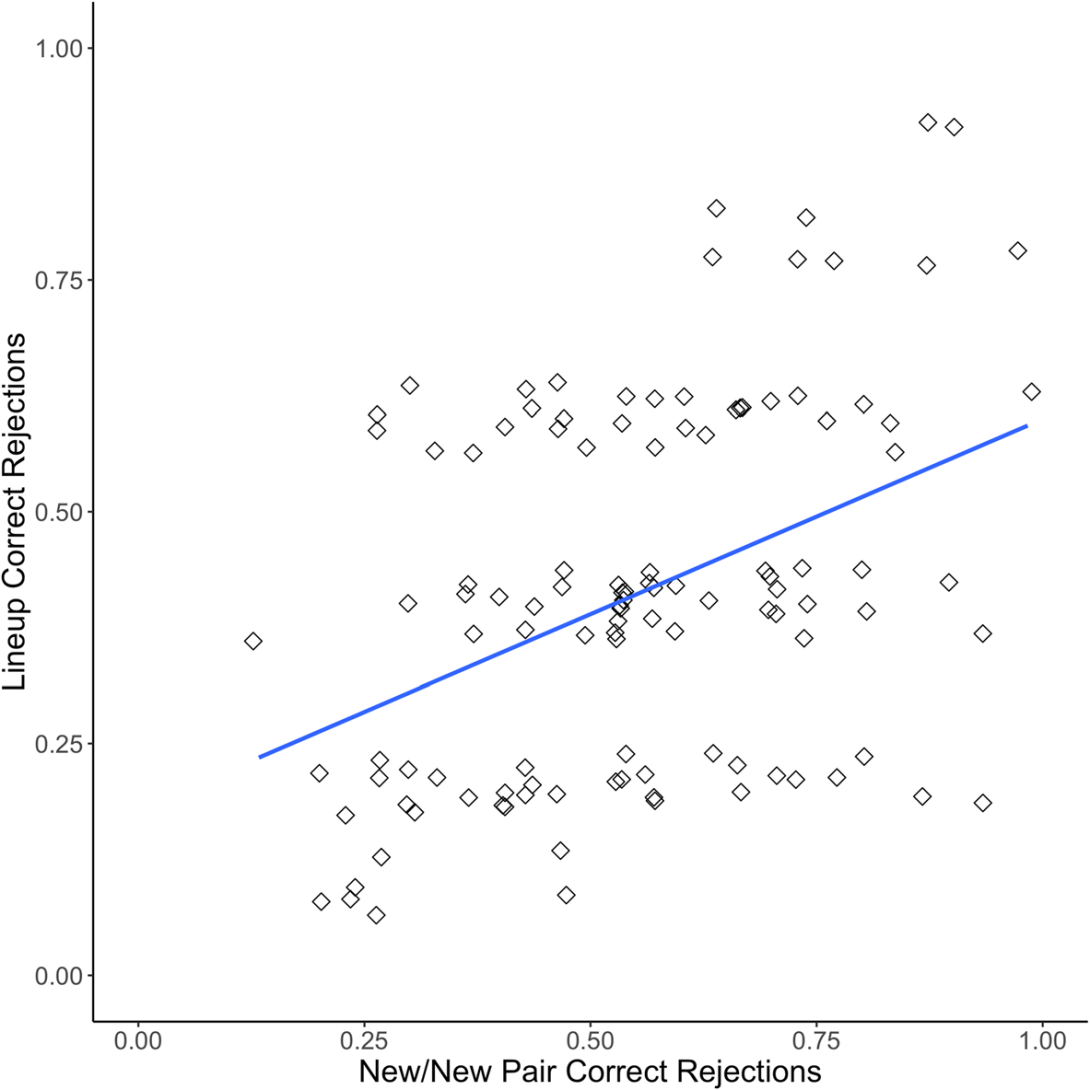
Proclivity to choose correlation for Experiment 2 with linear trendline, both axes jittered

**Fig. 5:**
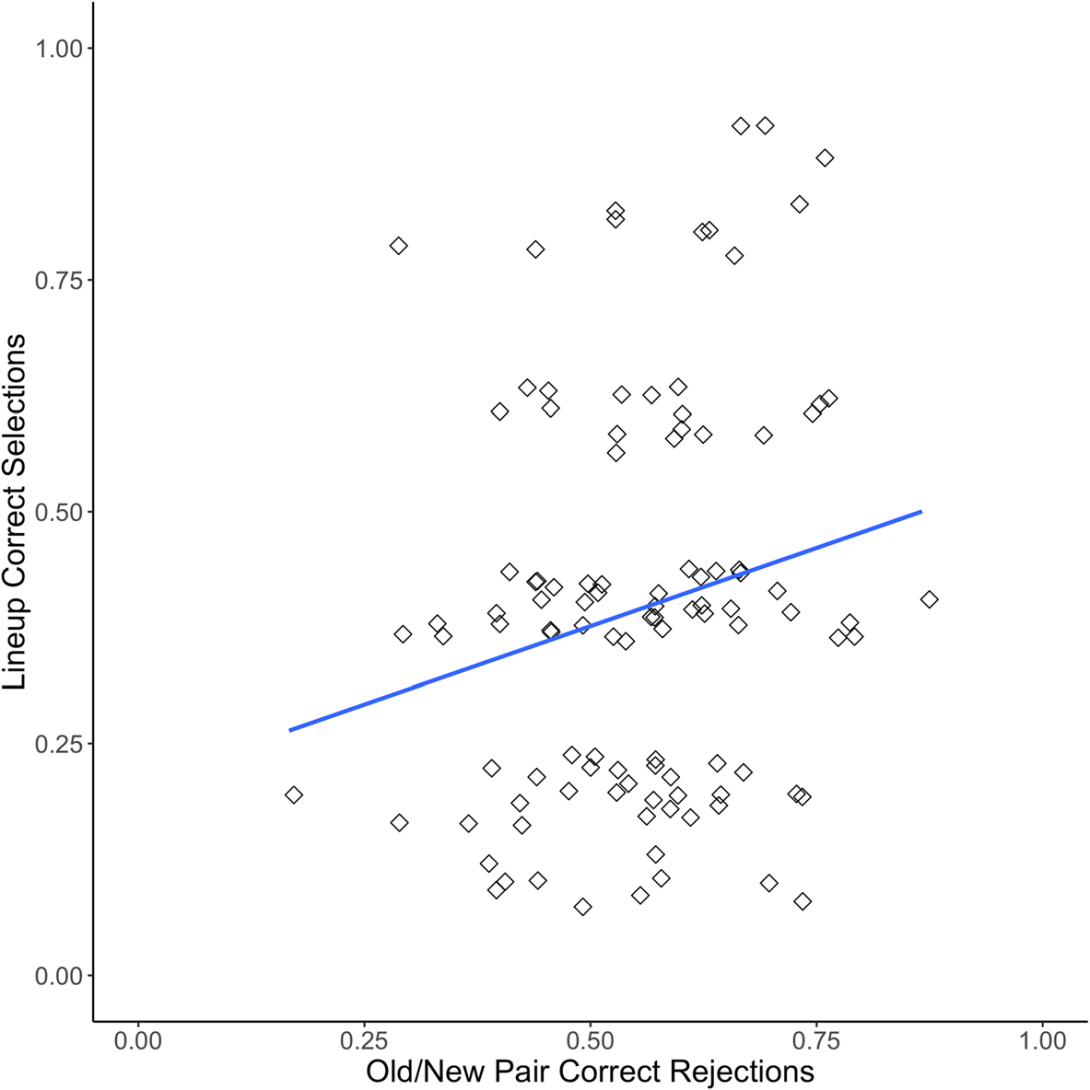
Face recognition skill correlation for Experiment 2 with linear trendline, both axes jittered

Figure 4 displays the proportion correct on new/new pairs and the proportion correct on CA lineups from Experiment 2 (*r*(113) = .42, *p* < .001, 95% CI [.26, .56]). Figure 5 displays the proportion correct on old/new pairs and the proportion correct on CP lineups for Experiment 2 (*r*(104) = .21, *p* = .031, 95% CI [.02, .39]).

### Discussion

Experiment 2 replicated the major findings from Experiment 1. The relationship between new/new pair correct rejection rates and CA lineup correction rejection rates was replicated and similar in strength to that of Experiment 1. The correlation between CP lineup accuracy and old/new pair accuracy also proved robust to a 2-day delay and to the presentation of lineups before the LST. The consistency of the test of the proclivity to choose across Experiments 1 and 2 suggests that its predictive utility for lineups is around *r* = .43. The results of Experiment 2 indicate that the predictive utility of the LST is robust to longer delays between viewing a crime and attending a lineup as well as to a procedural change that placed the lineup administration before the LST. These findings provide support for the potential utility of a test of proclivity to choose in applied settings.

## General discussion

Our findings add to a growing literature (Andersen et al., 2014; Bindemann et al., 2012; Geiselman et al., 2001; Morgan et al., 2007) supporting the idea that performance on standardized tests of face recognition may be a reliable index of individual differences in eyewitness ID performance. The sizes of the correlations are typical of those seen in other face recognition literature, particularly tests of the relationships of CFMT with various other face memory and perception tasks (Bobak et al., 2016; Bowles et al., 2009; McGugin et al., 2012; McKone et al., 2011). The main relationship, between proclivity to choose on the LST and rejection rates on lineups, may not have reached its upper bound in the present work. However, combining data into a larger set enables relatively stable prediction of the true strength of the correlations (Schönbrodt & Perugini, 2013). Collapsing across the two experiments reported here and the two accompanying pilot studies (see Additional files 1, 2, 3, 4 and 5 for descriptions of the pilot studies and scatterplots showing combined data), the strength of the proclivity to choose correlation was *r*(347) = .43, 95% CI [.34, .51] and the strength of the face recognition skill correlation was *r*(200) = .27, 95% CI [.13, .39]. The robustness of the correlation with proclivity to choose across the four samples suggests it has prospective utility to predict lineup decisions in the real world. The correlation between old/new rejection rates and CA lineup rejection rates in several samples is also a reflection of the strength of proclivity to choose as a stable individual difference and further supports the findings of Kantner and Lindsay (2012, 2014).

That proclivity to choose in face recognition has more predictive value for CA lineups than for CP lineups is a new finding, as most known predictors of eyewitness accuracy are more (if not exclusively) useful for CP lineups. Having predictors on both sides of being good at faces is desirable, given the uncertainty of the culprit’s presence in the real world. If this new measure proves to be reliable, a score for a witness could be used to weigh ID evidence. A lineup rejection should be considered more exonerating if the witness has a high proclivity to choose, while an ID from a witness with a low proclivity to choose would constitute strong evidentiary support that the suspect was the culprit.

Unlike real witnesses, our subjects watched several unrelated crime videos and attended a lineup for each video. While the use of multiple lineups was intended to increase the stability of our estimates of individuals’ lineup decision tendencies, most real-world witnesses attend a single lineup. To determine whether our use of multiple lineups affected the basic pattern of results relative to the use of only a single lineup, we calculated the correlation between the LST and performance on the very first lineup for each subject. In doing so, we combined data from the two experiments reported here and the two pilot studies reported in Additional File 1. Across all 347 participants for whom the first lineup was CA, 189 made false IDs on that lineup. For these choosers, the average new/new pair accuracy on the LST was .50, 95% CI [.46, .53]. For the remaining 158 participants who rejected that first CA, the average new/new pair accuracy on the LST was .60, 95% CI [.57, .63]. That difference was on the smaller side but statistically significant, and the same direction of difference was observed in each of the four experiments.

These initial data from our LST should be considered a preliminary step towards an eyewitness prediction measure of an individual’s response bias. We utilized standard laboratory procedures and emphasized the experimental control at this initial stage, but increased ecological validity in future work will be essential for establishing the usefulness of a test such as the LST in applied settings. Therefore, an important direction for future research is to conduct studies of the LST in more realistic conditions. In addition, changes to the materials or the procedure of the LST could alter (and perhaps strengthen) the relationship between proclivity to choose in a lineup and proclivity to choose in the LST. For example, the stimulus set we used in the LST contained more male than female faces, while test trials often contained one male and one female face. Gender biases may have added noise to our measurements of individuals’ choosing behavior, and future research could eliminate this possibility by using items with only one gender. On the other hand, the use of a more racially diverse face set would make the test more applicable for police precincts with diverse citizenship. In addition, while the facial expressions in the LST differed between study and test, they were homogeneous along many other dimensions. Faces that differ more markedly between presentation at study and at test (e.g., in viewing angle, brightness, or resolution) would better emulate the differences between the first and second exposures to a face in eyewitness situations and may increase the correspondence between decisions on the two tasks.

Performance on the LST was generally poor. Although it matched the average level of performance on the lineup task, it is an open question for how an LST yielding higher discrimination might correlate with lineup decisions. For example, accuracy on the LST was a weak predictor of lineup performance in the current experiments, but the relationship might be stronger if LST sensitivity was farther above chance.

Information about an individual witness’s likelihood of identifying innocent and guilty suspects could be combined with other measures of the witness’s performance, such as confidence or response latency. A complete model for weighing eyewitness ID decisions would also account for characteristics of the lineup (e.g., functional size), witnessing conditions (e.g., lighting, duration), and the delay between the witnessed event and the lineup, along with the prior odds that the suspect is the culprit (based on other aspects of crime-relevant evidence).

Finally, we know of no previously published study that presented both two-person mini-lineups and full-sized lineups to the same group of participants. That mini-lineups account for some variance in lineup scores but leave a substantial portion of the variance unaccounted for suggests researchers using mini-lineups as placeholders for real lineups should exercise caution when interpreting their results. Mini-lineups based on study phases with many trials are still useful, however, because it is critical to show a research participant many lineups to account for more of the variance in individual memory abilities.

## Conclusions

How individual differences work in eyewitness ID must receive more attention from researchers. We developed a two-alternative non-forced-choice face recognition test that reliably predicted an individual’s proclivity to choose in a series of lineups. Proclivity to choose may be an important facet of lineup decisions and could be of use to the police as part of a package of person- and situation-based predictors that jointly provide important information for weighing eyewitness evidence of the guilt or innocence of a suspect.

### Additional files

Additional file 1: The importance of decision bias for predicting eyewitness lineup choices: Toward a Lineup Skills Test. (DOCX 20 kb)

Additional file 2: **Figure S1.** Proclivity to choose correlation for Pilot Experiment 1 with linear trendline, both axes jittered. (PNG 229 kb)

Additional file 3: **Figure S2.** Proclivity to choose correlation for Pilot Experiment 2 with linear trendline, both axes jittered. (PNG 241 kb)

Additional file 4: **Figure S3.** Proclivity to choose correlation for all four experiments combined with linear trendlines, both axes jittered. (PNG 495 kb)

Additional file 5: **Figure S4.** Face recognition skill correlation for Experiments 1 and 2 combined with linear trendlines, both axes jittered. (PNG 344 kb)

## Appendix

### LST Study phase instructions

This study has to do with eyewitness suspect ID. Later, you will be shown five photo-spread lineups, one for each of the crime videos you just watched. As in the real-world, each lineup will include a suspect but it is possible that in one or more of the lineups the suspect will not be the culprit. The ideal witness identifies the culprit if present in the lineup, and rejects the lineup if the culprit is not present.

We are attempting to create a Lineup Skill Test. Our test has two steps. First, we will present a long series of faces of university students, one face at a time. Then you will take a test in which many pairs of faces will be presented (one pair at a time) and you will be asked to say which, if either, of the faces was on the study list. Please note that the faces used in this Lineup Skill Test are faces of students at our university—they have nothing to do with the faces you saw in the crime videos. Our hypothesis is that people who do well on our Lineup Skill Test (i.e., people who pick the right face if one of the faces in a test pair had been studied, and who reject the pair if neither of the faces had been studied) will also do well in the final phase of this study, in which the lineups for the crime videos will be presented.

## Abbreviations

BFRT: Benton Facial Recognition Test
CA: Culprit absent
CFMT: Cambridge Face Memory Test
CI: Confidence interval
CP: Culprit present
ID: Identification
LST: Lineup Skills Test
MTurk: Mechanical Turk
SD: Standard deviation
SDT: Signal detection theory

## Footnotes

1 In this article, we sometimes refer to face recognition ability or skill as analogous to the signal detection theory term “sensitivity.” Likewise, we often use the term “proclivity to choose” as analogous to the term “response bias.”

2 In Hosch’s lineups, participants were to identify the experimenter who had given them the task instructions. The results discussed here are collapsed across CP and CA lineups, as the report does not include group sizes.

3 Some investigators have described unpublished studies that seem to show effects of a similar size to those reported above (see Deffenbacher et al., 1978, in Table 2) that did not reach significance because they were underpowered. Deffenbacher et al. presented otherwise unpublished efforts to predict eyewitness accuracy at the *Practical Aspects of Memory Conference* in Cardiff (1978), in which an overall score on a yes/no face recognition test was not significantly correlated with accuracy on a very difficult lineup. Additionally, Hosch (1994) wrote that unpublished findings from Shepherd, Davies, and Ellis (1980) showed that recognition bias was predictive of eyewitness accuracy but sensitivity was not.

4 We thank L. L. Jacoby (personal communication, 2011) for suggesting the use of a two-alternative non-forced-choice test in this context.

5 As the photos were taken years ago, we did not predict the possibility of posting them online and thus, do not have consent to share the face set on the Open Science Framework. We will privately share the set upon request. See [https://osf.io/euchx/] for downloadable copies of our Qualtrics program.

6 Interested readers may take a version of our procedure at goo.gl/LnhBhr

7 We conducted additional exploratory analyses to assess whether the tendency to reject new/new pairs in the LST correlated with decisions on lineups when the culprit was present. Rejection rates on new/new pairs predicted rejection rates of CP lineups (*r*(92) = .29, *p* = .005, 95% CI [.09, .47]), a relationship roughly equal in magnitude to that between rejection rates on old/new pairs and CP lineup rejection rates (*r*(92) = .25, *p* = .015, 95% CI [.05, .43]). New/new rejection rates also predicted accuracy on CP lineups when participants chose a suspect (as opposed to rejecting the lineup; *r*(92) = .29, *p* < .005, 95% CI [.09, .47]).
