# Supplementary material for: The importance of decision bias for predicting eyewitness lineup choices: toward a Lineup Skills Test
Source: Cogn Res Princ Implic. 2019 Jan 28;4:2. doi: 10.1186/s41235-018-0150-3 (PMC6352739; doi:10.1186/s41235-018-0150-3)
Supplement: Supplementary file 1 — The importance of decision bias for predicting eyewitness lineup choices: Toward a Lineup Skills Test. (DOCX 20 kb) [file 41235_2018_150_MOESM1_ESM.docx]

**Additional File 1: The importance of decision bias for predicting eyewitness lineup choices: Toward a Lineup Skills Test**

**Pilot Experiment 1**

Pilot Experiment 1 was conducted with a procedure exactly the same as that of Experiment 1 in the main report of this work, but some critical methodological details are repeated here to enable standalone reading of these Supplementary Materials.

Participants first studied a large set of faces presented one at a time. The subsequent LST utilized a two-alternative non-forced-choice recognition task (meaning that participants could reject test pairs as unstudied) in which 50% of the trials contained a studied face and an unstudied face and the other 50% contained two unstudied faces.^^[[1]](#footnote-1)^^ By measuring accuracy when choosing on pairs containing one studied face and one non-studied face (target-present pairs), the LST provides a sensitivity-type measure of face recognition skill. By measuring rejection rates of pairs containing two non-studied faces (a target-absent pair), it provides a measure of proclivity to choose. We used face recognition skill and proclivity to choose scores to predict performance on five eyewitness identification lineups in each of four studies.

**Method**

**Participants.** Participants were recruited online via Amazon’s Mechanical Turk (*N* = 69) for $0.60. Following exclusionary criteria established before the data collection began, participants who confessed to major distractions or to skipping portions of the procedure were eliminated from the analysis (*N* = 2), as were participants who did not stay on the crime video pages long enough to watch them (*N* = 2). These exclusions were made before we analyzed the data. Data from the remaining 65 participants were used for analysis.

Participants self-reported demographics. The average reported age was 37.8 years, with a range from 20 to 71. The sample included 40 women, 58 native English-speakers, 33 who reported having attended some university courses, and 61 who reported having taken no university courses in psychology.

**Materials.** The five crime videos were clipped from British television crime dramas and depicted middle-aged Caucasian male culprits committing crimes (see the Wiki section of <https://osf.io/euchx/> for more information about the videos). Clips ranged from 47 to 83 seconds in length and were presented with the original sound tracks. Lineups each contained six individual photos about 250x350 pixels in size. Figure 1 shows each lineup, which consisted of men who fit a description of the culprit selected from the State of Florida’s online database of criminal mugshots. The photos were edited so that all members were wearing similar clothing. Similar excerpts from the same crime shows were used in unpublished experiments conducted as part of an undergraduate thesis at the University of Victoria by Byrona Tweedy (2011) under the supervision of the third author; we pre-designated as our innocent suspect the member of each CA lineup who had most often been selected in Tweedy's studies.

The photos for the face test were taken in front of a gray backdrop and showed head-and-shoulders views in color with a neutral expression. Photos were 600x600 pixels on screen, and all the people in the photos had no obviously distinctive features such as tattoos or scars. Faces were taken from our in-house face database.^^[[2]](#footnote-2)^^ The stimulus set contained 120 Caucasian faces (33 female). For the test phase, we gathered photos taken in the same session as those in the study phase but with the subject smiling (such that face recognition was tested rather than photo recognition; Bruce & Young, 1986).

**Procedure.** MTurk participants accepted the task on Amazon’s work exchange server and were linked to a survey hosted on Qualtrics, where they viewed the crime videos.^^[[3]](#footnote-3)^^ Next, participants studied a set of 30 digital photos of Caucasian faces for 1s each with a 1s gray mask between. Five pre-randomized photo sets were created such that they all contained differently ordered faces in the LST and a unique rotation of the order in which the five crimes were presented (see Mansour, Beaudry, & Lindsay, 2017, for discussion of the ecological validity of presenting multiple crimes and lineups). Participants were simply told to watch the videos and were not warned beforehand that the videos would depict crimes. After a 5-m distractor task, participants began our LST test phase, for which they were explicitly told our intentions. We aimed to be as open with participants as possible to avoid spontaneous creation of unintended demand characteristics; we also intended to retain applicability for a wide array of police precincts that may have differing rules regarding disclosures to witnesses.

The instructions explained the procedure in full and noted that the study had to do with eyewitness identification (see Appendix 1). After the study phase, participants moved on to the test, in which a pair of digital photos (450x450 pixels each) of faces appeared to the right and left of the mid-point of the screen in each of 60 trials. Half of the trials consisted of one studied or “old” face and one unstudied or “new” face; these constituted the face recognition skill portion of the test, in which the correct answer was either Right or Left. The other 30 trials each consisted of two unstudied faces; these constituted the proclivity to choose portion of the test, in which the correct answer was Neither. The two types of trials were randomly mixed. The first two and last two faces in the study list were not used in the test list to avoid primacy and recency effects. Test trials displayed selection options of Left, Neither, and Right that required a mouse click. Participants then rated confidence in each response on an 11-point scale (0-100). We then reminded participants of our aim to develop a Lineup Skills Test, and emphasized that a good witness chooses the criminal if he is present but also rejects a lineup from which the criminal is absent. Participants finished the procedure by completing five CA lineups. Crime and lineup order were counterbalanced and the face recognition study and test phases were presented in a fixed random order that was different for each version of the counterbalance.

**Results**

Individual accuracy rates on both the Lineup Skills Test and the lineups themselves have been converted to *z*-scores to facilitate comparison between studies, as they had varying delay lengths, different filler tasks, and different grand average accuracy rates. This practice does not inflate correlation coefficients; it in fact tends to reduce them slightly. Perfect scores and scores of zero have been changed to one-half the distance to the next possible score to enable *z*-scoring. See Table 3 for descriptive statistics of raw accuracy scores and [osf.io/euchx/] for participant average data. Z-scores were created using the NORMSINV function in Microsoft Excel, which returns a standardized value based on the inverse of the raw value (hence the correction for values of 0 and 1). The data are left in their raw form in graphs for readability. Figure A1 is a jittered scatterplot displaying proportion correct on New/New pairs and proportion correct on lineups for each subject. As predicted, the rate of correct rejections of New/New pairs predicted the rate of correct rejections of CA lineups, *r*(63)= .42, *p* < .001, 95% CI [.20, .60].

We also calculated some exploratory correlations from Pilot Experiment 1. A significant relationship was found between Old/New pair accuracy when choosing (correct answers divided only by incorrect selections of the wrong face, with pair rejections excluded) and the rate of correct rejections of lineups, *r*(63) = .33 *p* = .007, 95% CI [.09, .53], suggesting that face recognition sensitivity might be a predictor of lineup rejection ability. A relationship was discovered between Old/New pair rejection rate and lineup rejection rates, *r*(63) = .38, *p* = .002, 95% CI [.15, .57]. However, a hierarchical regression of lineup rejection rate treating New/New rejection rate as the predictor in model 1 did not benefit significantly from the addition of Old/New accuracy (correct responses divided by the sum of selections of the wrong face and incorrect pair rejections) and Old/New rejection rate as predictors in model 2, *F*(61) = 1.40, *p* = .599. Various correlations tested with confidence ratings did not reach significance.

Some correlations within the LST from Pilot Experiment 1 are also informative. Because they are within the same test, they are not *z*-scored. Rejection rate of New/New pairs was closely related to rejection rate of Old/New pairs, *r*(63) = .65, *p* < .001, 95% CI [.48, .77], which shows that response bias was highly related between the two trial types, further evidence of individual differences in response bias. Rejection rate of New/New pairs was also highly correlated with accuracy on Old/New pairs when choosing, *r*(63) = .64, *p* < .001, 95% CI [.47, .76].

**Pilot Experiment 2**

**Method**

**Participants**. An initial sample (*N* = 40) was collected from the University of Victoria (UVic) psychology participation pool, through which students earn extra credit points toward their psychology course in exchange for participation in studies. When the semester ended before we could hit the target *N* of 75*,* additional participants were recruited online via Amazon’s Mechanical Turk (*N* = 43) for $0.60. Following exclusionary criteria established in previous studies, no participants confessed to major distractions or to skipping portions of the procedure. Participants who confessed to having done a previous study of ours were removed (*N* = 2), as were those who answered correctly to less than half of immediate attention-check questions after the crime videos (*N* = 3). Two participants who recognized an actor from the Breaking & Entering video were also removed. Data from the remaining 36 MTurk participants and all 40 UVic participants were used for analysis.

MTurk participants self-reported demographics. The average reported age was 41.9 years, with a range from 21 to 65. The sample included 25 women, 31 native English-speakers, 25 who reported having earned at least a bachelor’s degree, and 10 who reported having taken no university courses in psychology.

**Materials and Procedure**. During the first session, participants were recruited and sent to a survey site where they watched the five crime videos. They then left information to be contacted in 48 hours to finish the procedure. Participants tested in the lab were scheduled in sessions 48 hours after their first; M. Turk participants observed delay lengths between 3 and 76 hours, with an average of 43.6 hours. The second session began with lineups for each crime in order to avoid the interference caused by the faces studied for the Lineup Skills Test, which now followed the lineup task. We used only CA lineups (proclivity to choose) in the current procedure.

**Results**

Figure A2 displays proportion correct on New/New pairs and proportion correct on CA lineups from Pilot Experiment 2, *r*(74)= .41, *p* < .001, 95% CI [.20, .58]. Old/New pair accuracy when choosing was again correlated with CA lineup rejection rates, *r*(74) = .30, *p* < .01, 95% CI [.08, .49]. Finally, the relationship between Old/New pair rejection rate and CA lineup rejection rates was again significant, *r*(74) = .27, *p* < .05, 95% CI [.05, .47].

Figure A3 displays proportion correct on New/New pairs and proportion correct on CA lineups across all four samples, and Figure A4 displays proportion correct on Old/New pairs and proportion correct on CP lineups across the two samples that included CP lineups in the procedure.

1. We thank Larry L. Jacoby (Personal Communication, 2011) for suggesting the use of a two-alternative non-forced-choice test in this context. [↑](#footnote-ref-1)
2. As the photos were taken years ago, we did not predict the possibility of posting them online and thus do not have consent to share the face set on the Open Science Framework. We will privately share the set upon request. See [https://osf.io/euchx/] for downloadable copies of our Qualtrics programs. [↑](#footnote-ref-2)
3. Interested readers may take a version of our survey at goo.gl/LnhBhr [↑](#footnote-ref-3)
