# Supplementary figures and images for: The importance of decision bias for predicting eyewitness lineup choices: toward a Lineup Skills Test
Source: Cogn Res Princ Implic. 2019 Jan 28;4:2. doi: 10.1186/s41235-018-0150-3 (PMC6352739; doi:10.1186/s41235-018-0150-3)

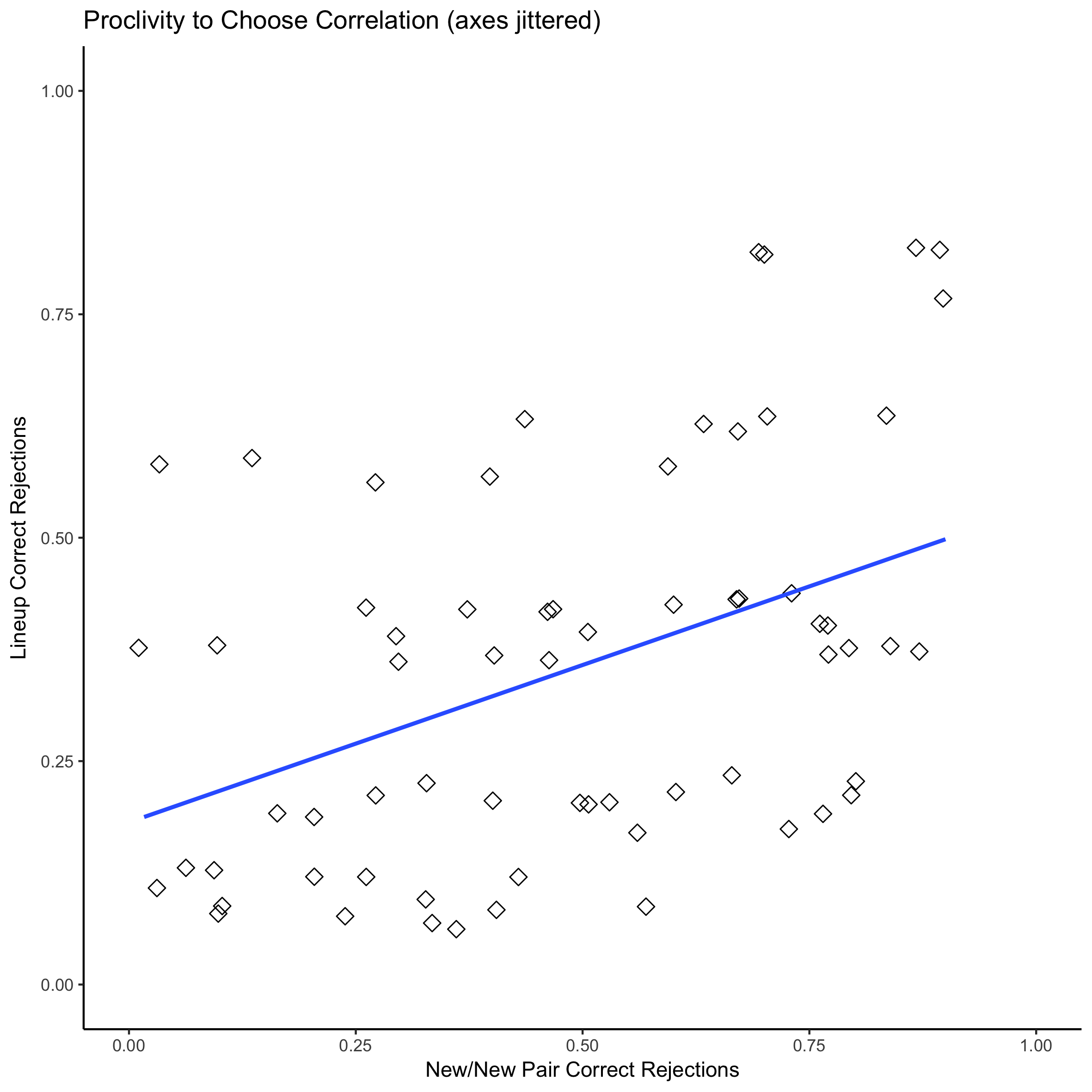

Supplement: Supplementary file 2 — Figure S1. Proclivity to choose correlation for Pilot Experiment 1 with linear trendline, both axes jittered. (PNG 229 kb) [file 41235_2018_150_MOESM2_ESM.png]

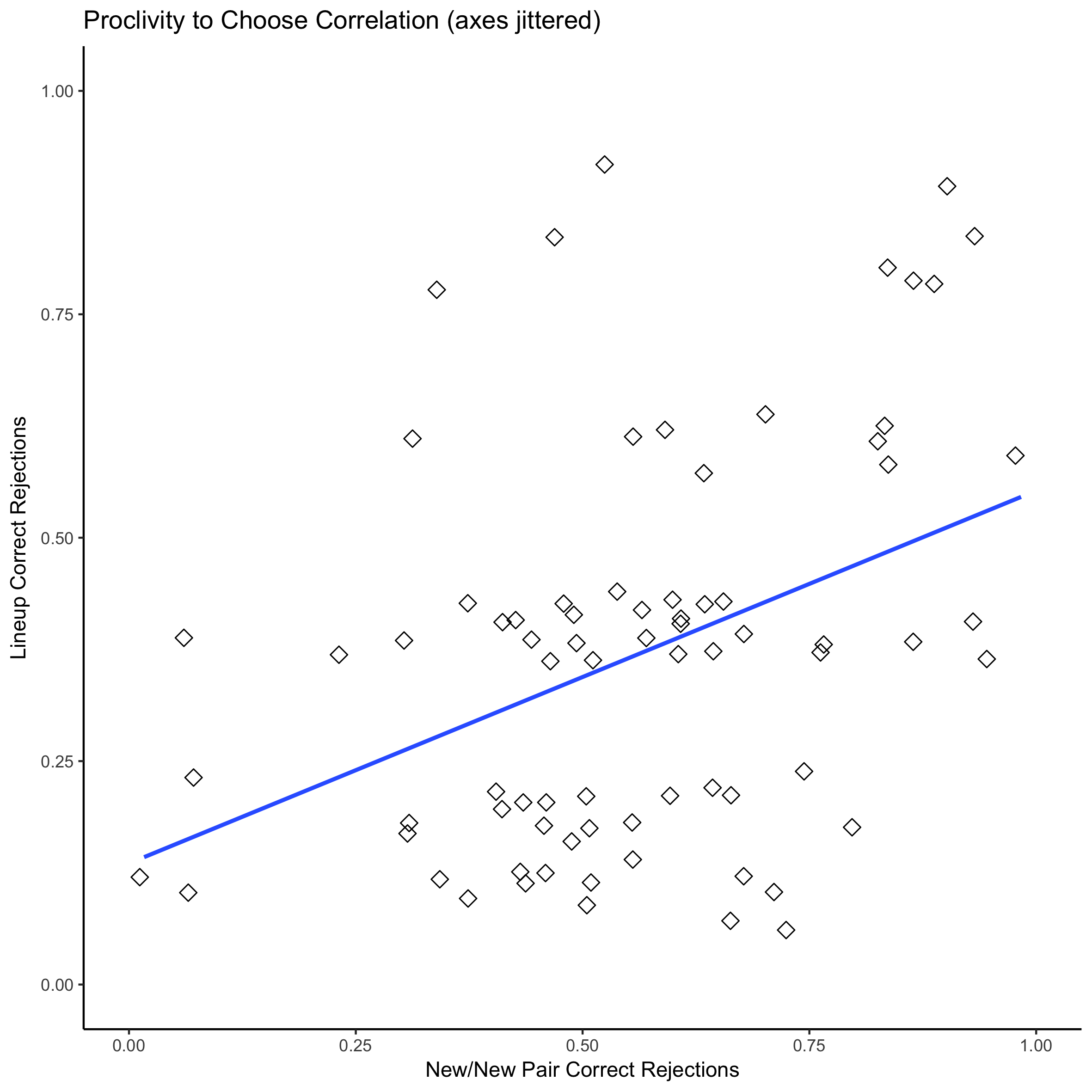

Supplement: Supplementary file 3 — Figure S2. Proclivity to choose correlation for Pilot Experiment 2 with linear trendline, both axes jittered. (PNG 241 kb) [file 41235_2018_150_MOESM3_ESM.png]

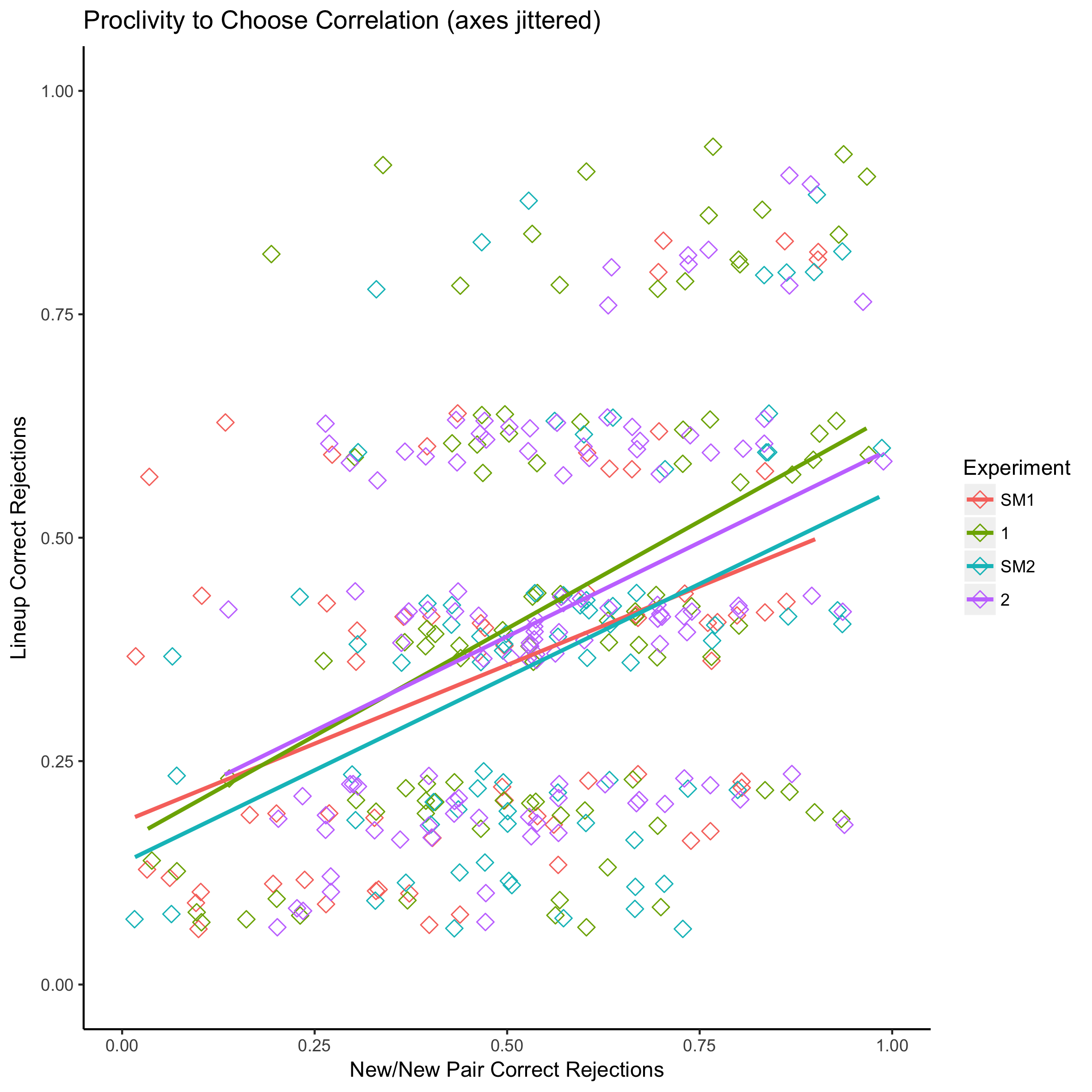

Supplement: Supplementary file 4 — Figure S3. Proclivity to choose correlation for all four experiments combined with linear trendlines, both axes jittered. (PNG 495 kb) [file 41235_2018_150_MOESM4_ESM.png]

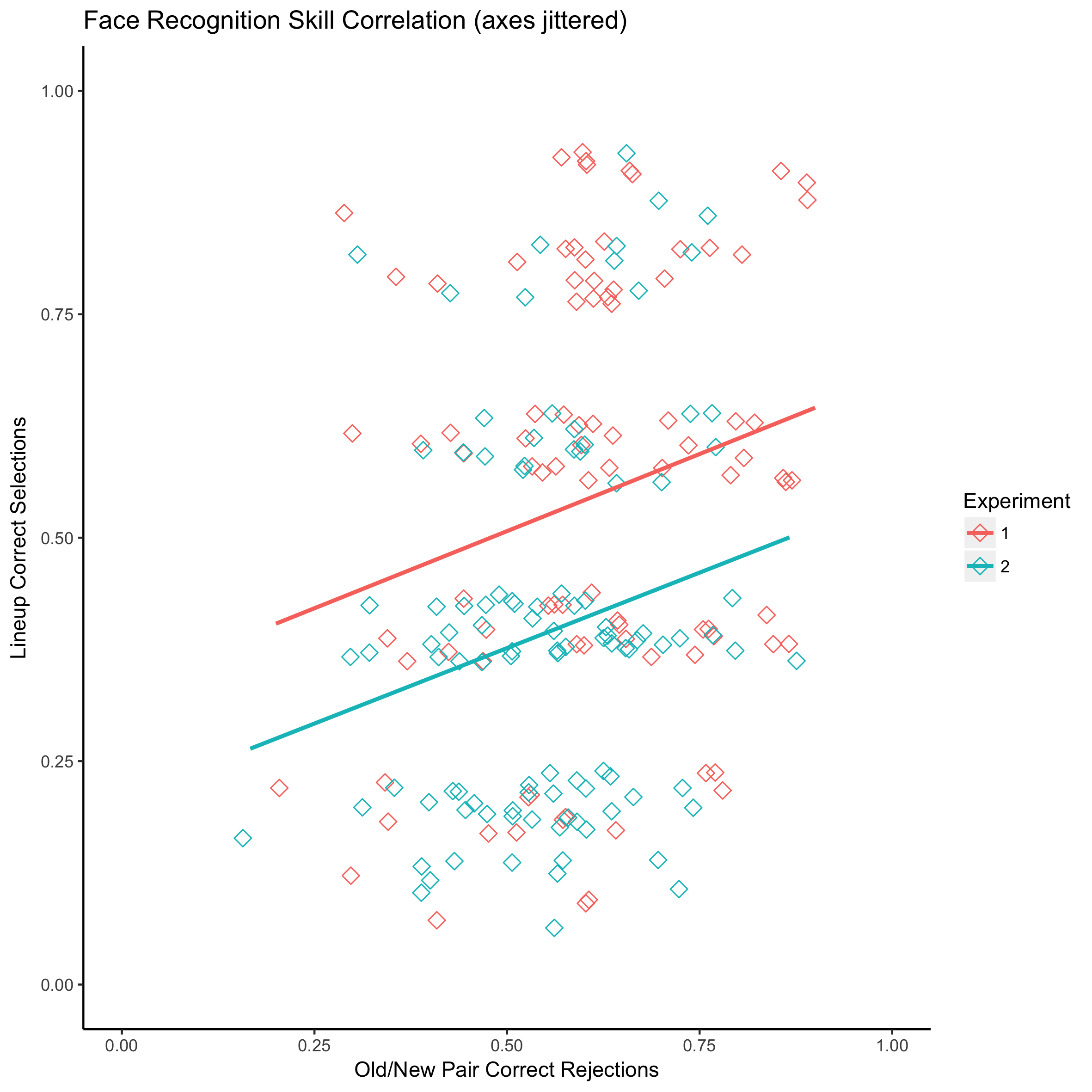

Supplement: Supplementary file 5 — Figure S4. Face recognition skill correlation for Experiments 1 and 2 combined with linear trendlines, both axes jittered. (PNG 344 kb) [file 41235_2018_150_MOESM5_ESM.png]
